# Supplementary material for: ACE2-independent sarbecovirus cell entry can be supported by TMPRSS2-related enzymes and can reduce sensitivity to antibody-mediated neutralization
Source: PLoS Pathog. 2024 Nov 13;20(11):e1012653. doi: 10.1371/journal.ppat.1012653 (PMC11559990; doi:10.1371/journal.ppat.1012653)
Supplement: S1 Table — (DOCX) [file ppat.1012653.s001.docx]

**Supplemental table 1: Information on the spike proteins under study**

| Spike | Virus | Identifier | RBD clade | Host | Region |
| --- | --- | --- | --- | --- | --- |
| SARS-2-S | Human SARS-CoV-2 hCoV-19/Wuhan/Hu-1/2019 | GISAID: EPI_ISL_402125 | 1b | Human (*Homo sapiens*) | Asia (China) |
| RaTG13-S | Bat SARSr-CoV hCoV-19/bat/Yunnan/RaTG13/2013 | GISAID: EPI_ISL_402131 | 1b | Bat (*Rhinolophus affinis*) | Asia (China) |
| P5L-S | Pangolin SARSr-CoV hCoV-19/pangolin/Guangxi/P5L/2017 | GISAID: EPI_ISL_410540 | 1b | Malayan pangolin (*Manis javanica*) | Asia (China) |
| cDNA8-S | Pangolin SARSr-CoV hCoV-19/pangolin/Guangdong/cDNA8-S/2019 | GISAID: EPI_ISL_471461 | 1b | Malayan pangolin (*Manis javanica*) | Asia (China) |
| Rs4081-S | Bat SARSr-CoV Rs4081 | GenBank: KY417143.1 | 2 | Bat (*Rhinolophus sinicus*) | Asia (China) |
| Rs4237-S | Bat SARSr-CoV RS4237 | GenBank: KY417147.1 | 2 | Bat (*Rhinolophus sinicus*) | Asia (China) |
| SARS-1-S | Human SARS-CoV-1/Frankfurt-1 | GenBank: AY291315.1 | 1a | Human (*Homo sapiens*) | Europe (Germany) |
| WIV1-S | Bat SARSr-CoV WIV1 | GenBank: KF367457.1 | 1a | Bat (*Rhinolophus sinicus*) | Asia (China) |
| LYRa11-S | Bat SARSr-CoV LYRa11 | GenBank: KF569996.1 | 1a | Bat (*Rhinolophus affinis*) | Asia (China) |
| RsSHC014-S | Bat SARSr-CoV RsSHC014 | GenBank: KC881005.1 | 1a | Bat (*Rhinolophus sinicus*) | Asia (China) |
| Rs4231-S | Bat SARSr-CoV Rs4231 | GenBank: KY417146.1 | 1a | Bat (*Rhinolophus sinicus*) | Asia (China) |
| Rs4874-S | Bat SARSr-CoV Rs4874 | GenBank: KY417150.1 | 1a | Bat (*Rhinolophus sinicus*) | Asia (China) |
| Rs7327-S | Bat SARSr-CoV Rs7327 | GenBank: KY417151.1 | 1a | Bat (*Rhinolophus sinicus*) | Asia (China) |
| BM48-31-S | Bat SARSr-CoV BM48-31/BGR/2008 | GenBank: GU190215.1 | 3 | *Rhinolophus blasii* | Europe (Bulgaria) |
