# Supplementary material for: ACE2-independent sarbecovirus cell entry can be supported by TMPRSS2-related enzymes and can reduce sensitivity to antibody-mediated neutralization
Source: PLoS Pathog. 2024 Nov 13;20(11):e1012653. doi: 10.1371/journal.ppat.1012653 (PMC11559990; doi:10.1371/journal.ppat.1012653)
Supplement: S2 Table — (DOCX) [file ppat.1012653.s002.docx]

**Supplemental table 2: Information on the cell lines used**

| **Cell line** | **Species** | **Organ** | **Modification** | **Culture medium** |
| --- | --- | --- | --- | --- |
| Vero | African green monkey (*Cercopithecus aethiops*) | Kidney | n.a. | DMEM + 10% FCS + Pen/Strep |
| Vero-ACE2+TMPRSS2 | African green monkey (*Cercopithecus aethiops*) | Kidney | Stable expression of human ACE2 and human TMPRSS2 | DMEM + 10% FCS + Pen/Strep + Blasticidin (2 µg/ml) + Puromycin (1 µg/ml) |
| Vero-TMPRSS2 | African green monkey (*Cercopithecus aethiops*) | Kidney | Stable expression of human TMPRSS2 | DMEM + 10% FCS + Pen/Strep + Blasticidin (2 µg/ml) |
| MyDauLu/47 | Bat (*Myotis daubentonii*) | Lung | n.a. | DMEM + 10% FCS + Pen/Strep |
| PipNi/3 | Bat (*Pipistrellus pipistrellus*) | Kidney | n.a. | DMEM + 10% FCS + Pen/Strep |
| Caco-2 | Human (*Homo sapiens*) | Intestine | n.a. | MEM + 10% FCS 1% NEA + 10 mM sodium pyruvate + Pen/Strep + Puromycin (1 µg/ml) |
| 293T | Human (*Homo sapiens*) | Kidney | n.a. | DMEM + 10% FCS + Pen/Strep |
| 293T-ACE2 | Human (*Homo sapiens*) | Kidney | Stable expression of human ACE2 | DMEM + 10% FCS + Pen/Strep + Puromycin (1 µg/ml) |
| Huh-7 | Human (*Homo sapiens*) | Liver | n.a. | DMEM + 10% FCS + Pen/Strep |
| Li7 | Human (*Homo sapiens*) | Liver | n.a. | DMEM + 10% FCS + Pen/Strep |
| A549-ACE2 | Human (*Homo sapiens*) | Lung | Stable expression of human ACE2 | DMEM/F-12 + 10% FCS + Pen/Strep + Puromycin (1 µg/ml) |
| A549-ACE2+TMPRSS2 | Human (*Homo sapiens*) | Lung | Stable expression of human ACE2 and human TMPRSS2 | DMEM/F-12 + 10% FCS + Pen/Strep + Blasticidin (2 µg/ml) + Puromycin (1 µg/ml) |
| Calu-3 | Human (*Homo sapiens*) | Lung | n.a. | DMEM/F-12 + 10% FCS 1% NEA + 10 mM sodium pyruvate + Pen/Strep |
| Calu-3-ACE2 | Human (*Homo sapiens*) | Lung | Stable expression of human ACE2 | DMEM/F-12 + 10% FCS 1% NEA + 10 mM sodium pyruvate + Pen/Strep + Puromycin (1 µg/ml) |
| NCI-H522 | Human (*Homo sapiens*) | Lung | n.a. | RPMI + 10% FCS 1% NEA + 10 mM sodium pyruvate + Pen/Strep |
| BHK-21 | Syrian golden hamster (*Mesocricetus auratus*) | Kidney | n.a. | DMEM + 10% FCS + Pen/Strep |
