# Supplementary material for: ACE2-independent sarbecovirus cell entry can be supported by TMPRSS2-related enzymes and can reduce sensitivity to antibody-mediated neutralization
Source: PLoS Pathog. 2024 Nov 13;20(11):e1012653. doi: 10.1371/journal.ppat.1012653 (PMC11559990; doi:10.1371/journal.ppat.1012653)
Supplement: S3 Table — (DOCX) [file ppat.1012653.s003.docx]

**Supplemental table 3**

| **Plasma from convalescent donors** | | | | | | | |
| --- | --- | --- | --- | --- | --- | --- | --- |
| ID | Sex | Age (years) | Infected | Vaccinated | Vaccine | Time since infection (days) | Anti-S1 IgG (BAU/ml) |
| SI15 | M | 65 | yes | no | n.a. | unknown | unknown |
| SI18 | F | 74 | yes | no | n.a. | unknown | unknown |
| SI20 | M | 61 | yes | no | n.a. | unknown | unknown |
| SI22 | F | 25 | yes | no | n.a. | unknown | unknown |
| SI23 | F | 69 | yes | no | n.a. | unknown | unknown |
| SI24 | M | 61 | yes | no | n.a. | unknown | unknown |
| SI27 | M | 52 | yes | no | n.a. | unknown | unknown |
| SI33 | M | 75 | yes | no | n.a. | unknown | unknown |
| SI51 | M | 71 | yes | no | n.a. | unknown | unknown |
|  |  |  |  |  |  |  |  |
| **Plasma from 2x vaccinated donors** | | | | | | | |
| ID | Sex | Age (years) | Infected | Vaccinated | Vaccine | Time since last vaccination (days) | Anti- S1 IgG (BAU/ml) |
| 4847 | F | 57 | no | yes | BNT/BNT | 26 | 381 |
| 4848 | M | 29 | no | yes | BNT/BNT | 26 | 1221 |
| 4849 | M | 39 | no | yes | BNT/BNT | 24 | 2032 |
| 4863 | F | 58 | no | yes | BNT/BNT | 26 | 618 |
| 4865 | M | 57 | no | yes | BNT/BNT | 25 | 747 |
| 4867 | F | 52 | no | yes | BNT/BNT | 30 | 1529 |
| 4868 | F | 56 | no | yes | BNT/BNT | 30 | 392 |
| 4872 | F | 37 | no | yes | BNT/BNT | 25 | 1164 |
| 4877 | F | 29 | no | yes | BNT/BNT | 28 | 1131 |
|  |  |  |  |  |  |  |  |
| **Plasma from 3x vaccinated donors** | | | | | | | |
| ID | Sex | Age (years) | Infected | Vaccinated | Vaccine | Time since last vaccination (days) | Anti- S1 IgG (BAU/ml) |
| 8624 | F | 31 | no | yes | AZ/BNT/BNT | 123 | 2629 |
| 8631 | F | 61 | no | yes | AZ/BNT/BNT | 123 | 5429 |
| 8632 | F | 46 | no | yes | AZ/BNT/BNT | 123 | 2374 |
| 8639 | F | 50 | no | yes | AZ/BNT/BNT | 110 | 5576 |
| 8645 | F | 57 | no | yes | AZ/BNT/BNT | 134 | 8758 |
| 8649 |  | 53 | no | yes | AZ/BNT/BNT | 128 | 7828 |
| 8663 | F | 54 | no | yes | AZ/BNT/BNT | 127 | 2644 |
| 8664 | M | 51 | no | yes | AZ/BNT/BNT | 129 | 4819 |
| 8700 | F | 29 | no | yes | AZ/BNT/BNT | 136 | 3610 |
| 8701 | M | 33 | no | yes | AZ/BNT/BNT | 134 | 8194 |
|  |  |  |  |  |  |  |  |
| **Plasma from 4x vaccinated donors** | | | | | | | |
| ID | Sex | Age (years) | Infected | Vaccinated | Vaccine | Time since last vaccination (days) | Anti- S1 IgG (BAU/ml) |
| 9474 | F | 54 | no | yes | unknown/XBB | 33 | 2629 |
| 9476 | F | 51 | no | yes | unknown/XBB | 33 | 5429 |
| 9477 | M | 52 | no | yes | unknown/XBB | 33 | 2374 |
| 9479 | M | 62 | no | yes | unknown/XBB | 33 | 5576 |
| 9481 | F | 58 | no | yes | unknown/XBB | 23 | 8758 |
| 9484 | M | 45 | no | yes | unknown/XBB | 33 | 7828 |
| 9488 | Ma | 54 | no | yes | unknown/XBB | 26 | 2644 |
| 9493 | F | 61 | no | yes | unknown/XBB | 28 | 4819 |
| 9494 | F | 50 | no | yes | unknown/XBB | 33 | 3610 |
| 9496 | M | 40 | no | yes | BNT/BNT/  MOD/XBB | 33 | 8194 |

n.a.: not applicable

BNT: BNT162b2, XBB: BNT162b2 omicron XBB.1.5, AZ: Vaxzevria; MOD: Spikevax
