# Supplementary figures and images for: ACE2-independent sarbecovirus cell entry can be supported by TMPRSS2-related enzymes and can reduce sensitivity to antibody-mediated neutralization
Source: PLoS Pathog. 2024 Nov 13;20(11):e1012653. doi: 10.1371/journal.ppat.1012653 (PMC11559990; doi:10.1371/journal.ppat.1012653)

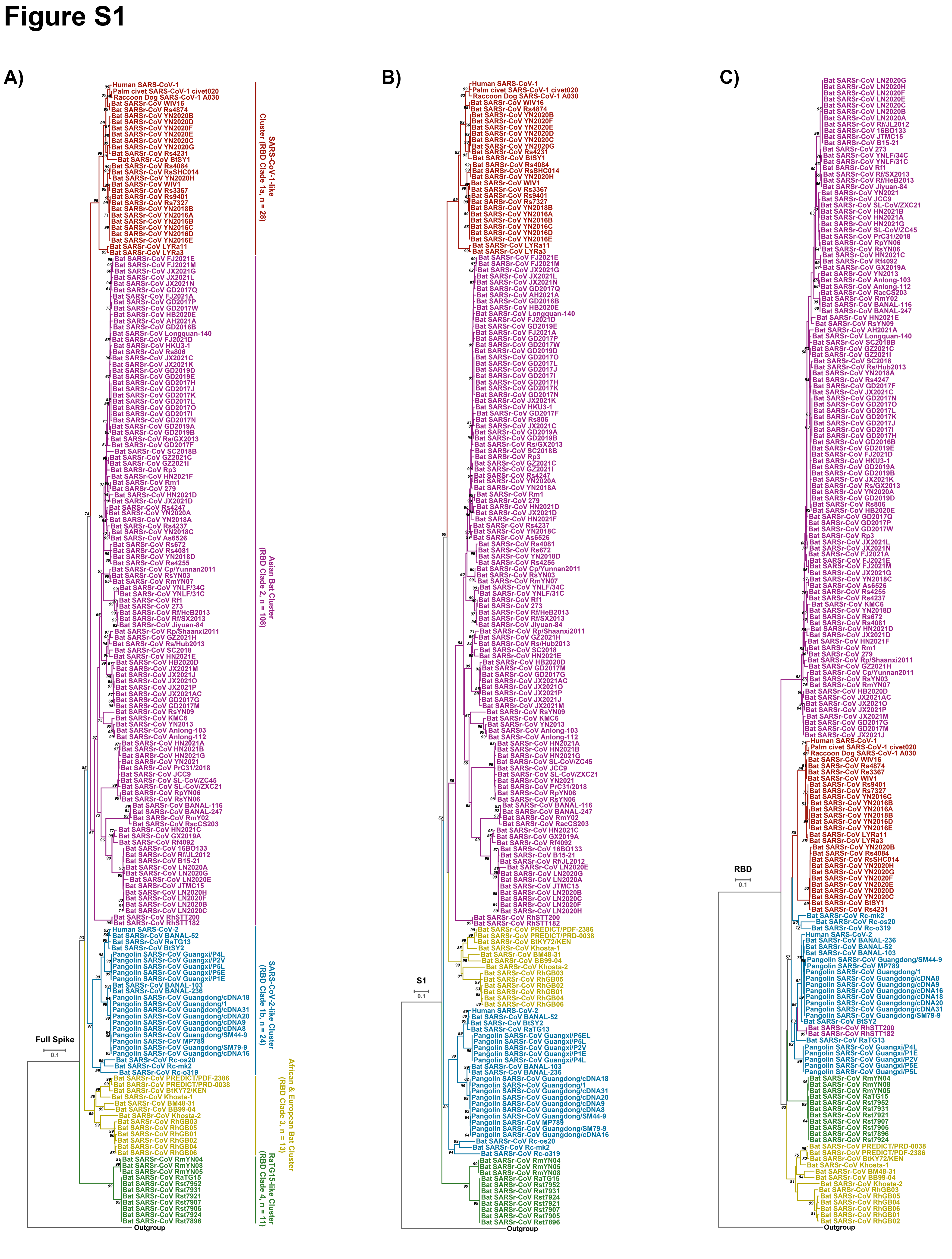

Supplement: S1 Fig — Phylogenetic analysis was based on full S amino acid sequence (A), S1 subunit (B) or RBD (C). (TIF) [file ppat.1012653.s004.tif]

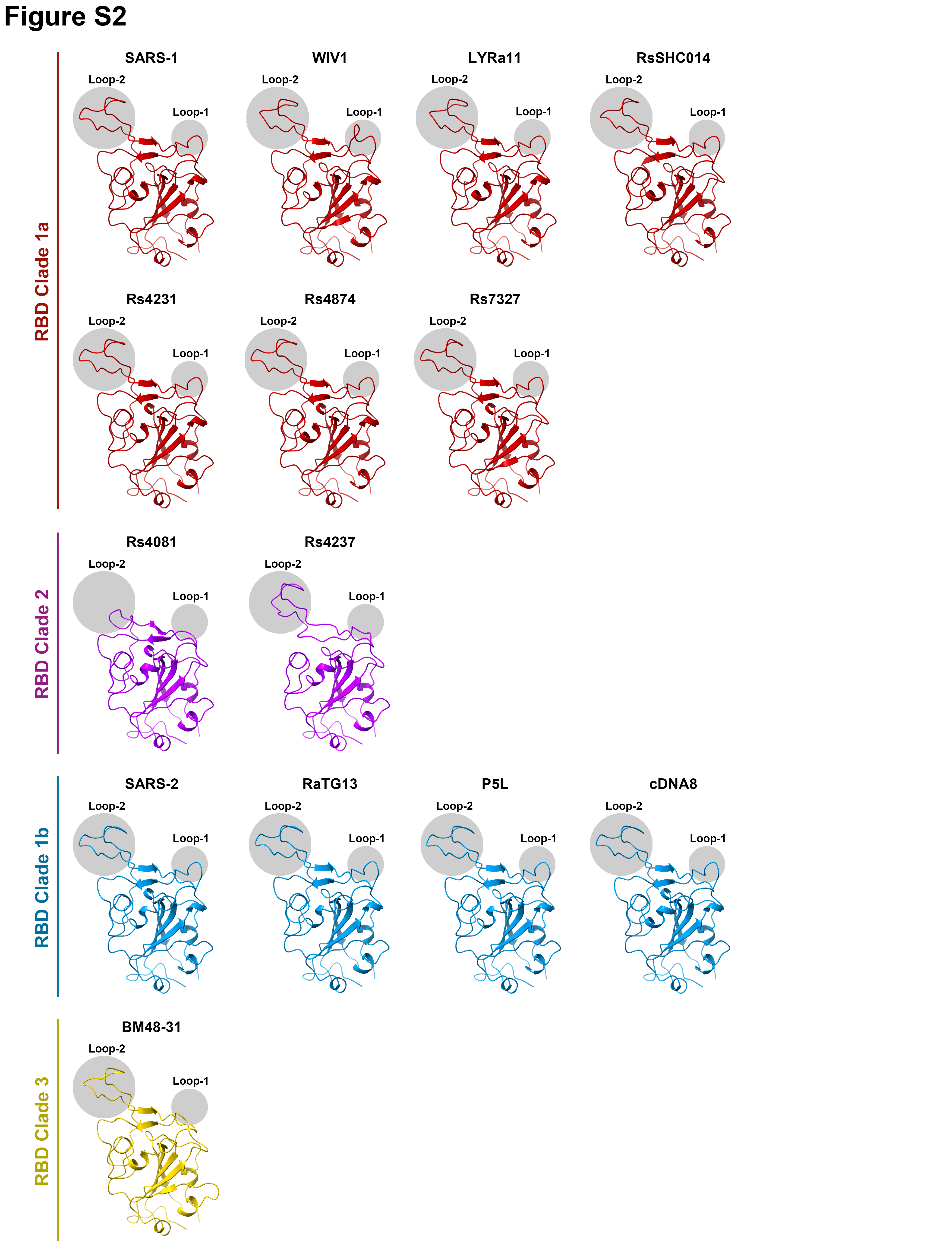

Supplement: S2 Fig — Individual models of the RBD structures presented in Fig 1B. (TIF) [file ppat.1012653.s005.tif]

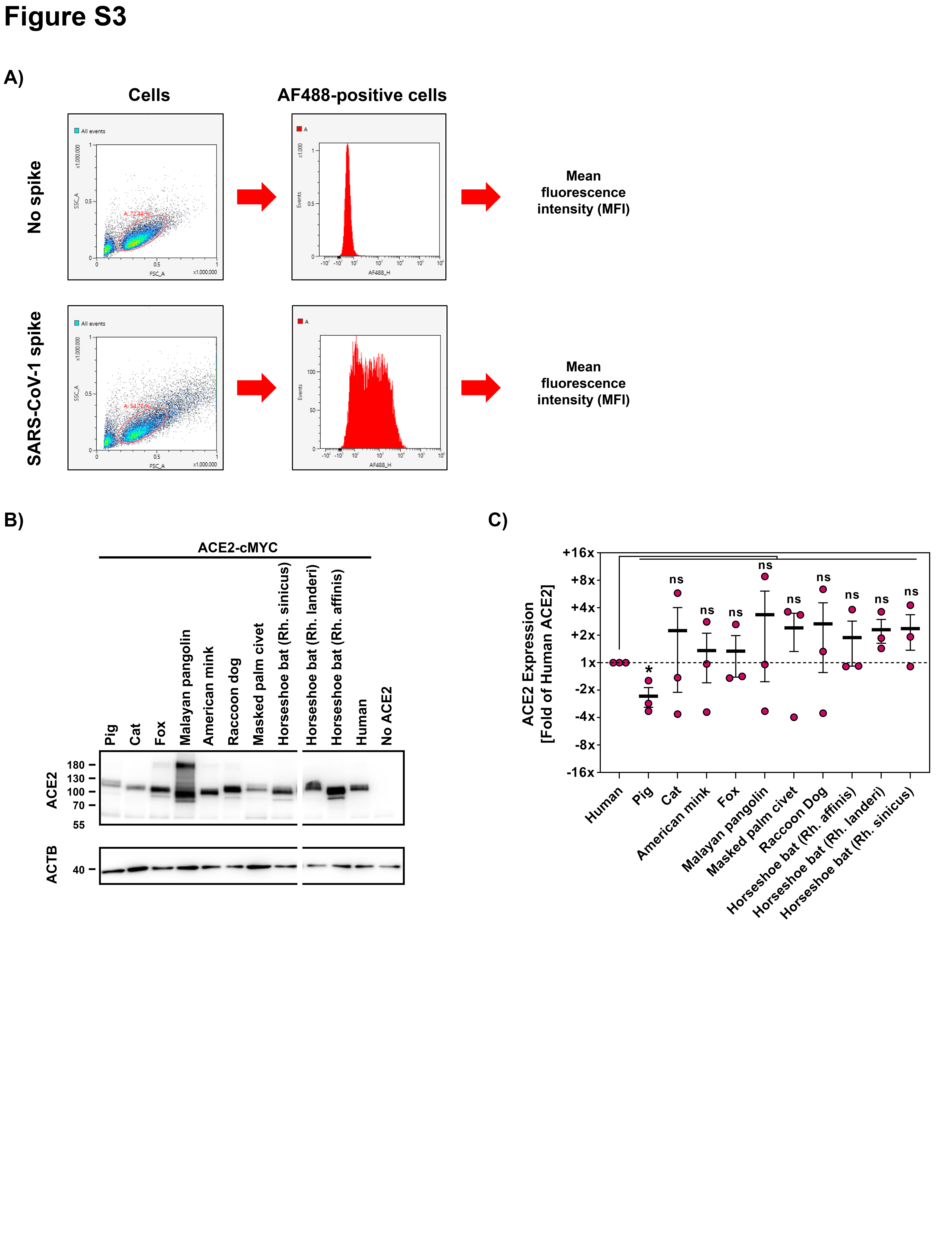

Supplement: S3 Fig — A) Gating strategy for flow cytometry (related to Fig 2A). The single cells were gated based on SSC-A and FSC-A, and the mean fluorescence intensity (MFI) of AF488-positive cells was selected for further analysis. B) Expression level of ACE2 orthologues (related to Fig 2B and 2C). BHK-21 cells were transfected with plasmids encoding ACE2 orthologues harboring a C-terminal c-myc epitop tag. At 48 h posttransfection, cell lysates were prepared and analyzed for ACE2 expression by SDS-PAGE and immunoblot using an anti-c-myc primary antibody and a corresponding peroxidase-coupled secondary antibody. Detection of ß-actin (ACTB) served as loading control. The results of a single experiment are shown and results were confrimed in two additional experiments (irrelevant lanes have been removed). Numerical values on the left indicate the molecular weight in kilodalton. C) Quantification of ACE2 orthologue expression. Presented are the average (mean) data from three biological replicates (each conducted with single samples). For normalization, ACE2 signals were first corrected for potential differences in sample loading by normalization against their respective ACTB signals and subsequently expression of animal ACE2 orthologues was normalized to human ACE2 (set as 1). Error bars indicate SEM. Statistical significance was assessed by two-tailed Student’s t-tests (p > 0.05, not significant [ns]; p ≤ 0.05, *). (TIF) [file ppat.1012653.s006.tif]

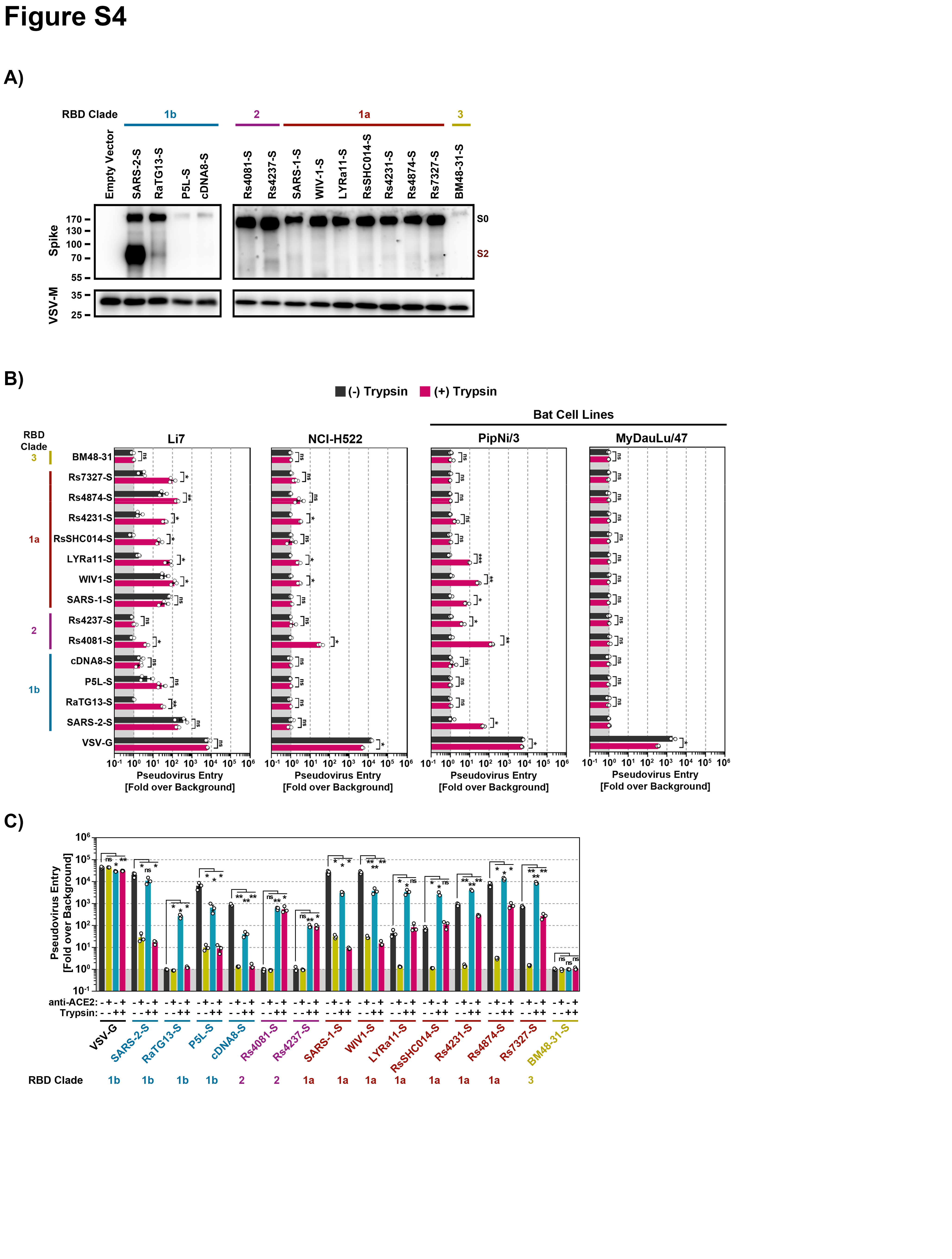

Supplement: S4 Fig — A) Incorporation of S proteins into VSV particles. Pseudoviruses bearing the indicated S proteins were concentrated by high-speed centrifugation through a sucrose cushion. Next, particles were lysed and S protein incorporation analyzed by SDS-PAGE immunoblot using anti-SARS-CoV-2 S2 and anti-VSV-M (loading control) primary antibodies, and the corresponding peroxidase-coupled secondary antibodies. The results of a representative experiment are shown and were confirmed in two additional experiments. Numerical values on the left indicate the molecular weight in kilodalton. B) Entry of S protein bearing particles into cell lines. Particles bearing the indicated S proteins (or no S protein) were preincubated with or without trypsin before being added to the respective cell lines. S protein-driven cell entry was analyzed by measuring the activity of virus-encoded firefly luciferase in the cell lysate at 16-18h post inoculation. Presented are the average of (mean) data from three biological replicates (each conducted with four technical replicates) in which cell entry was normalized against that measured for particles bearing no S protein (set as 1). Error bars show the SEM. Statistical significance was assessed by two-tailed Student’s t-tests (p > 0.05, not significant [ns]; p ≤ 0.05, *; p ≤ 0.01, **; p ≤ 0.001, ***). C) Blockade of S protein-driven cell entry by an anti-ACE2 antibody. Vero-TMPRSS2 cells were pre-incubated with anti-ACE2 antibody. Particles bearing the indicated S proteins were incubated with trypsin (50 μg/ml, 30 min, 37°C) followed by incubation with trypsin inhibitor (200 μg/ml, 10 min, 37°C) before addition onto target cells. S-protein-driven cell entry was analyzed by and data presented as described for panel A. Presented are the average (mean) data of three biological replicates, each performed with four technical replicates. Error bars show SEM. Statistical significance was assessed by two-tailed Student’s t-tests (p > 0.05, not significant [ns]; p [file ppat.1012653.s007.tif]

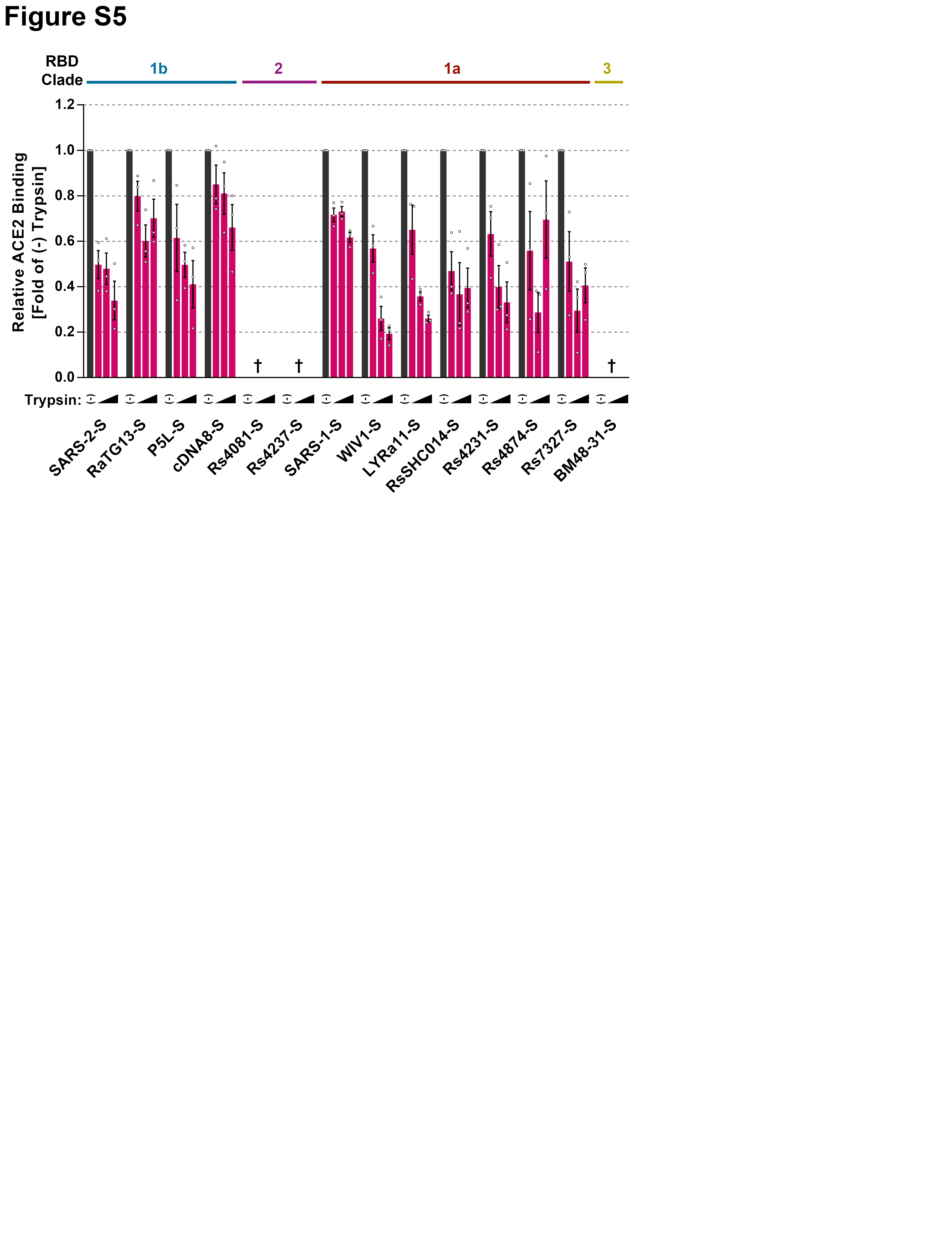

Supplement: S5 Fig — Binding of soluble human ACE2 to S protein expressing cells. 293T cells transiently expressing the indicated S proteins (or no S protein) were pre-incubated with different concentrations of trypsin or mock incubated. Thereafter, samples were incubated with soluble ACE2 containing a C-terminal Fc-tag (derived from human immunoglobulin G; solACE2-Fc) and subsequently incubated with an AlexaFluor-488-coupled secondary antibody. Finally, solACE2-Fc binding was analyzed by flow cytometry. Presented are the average (mean) data from three biological replicates (each conducted with single samples) in which solACE2-Fc binding to S protein expressing cells was normalized to binding to cells that were not treated with trypsin (set as 1). Error bars indicate SEM. (TIF) [file ppat.1012653.s008.tif]
